# Determination of Prolactin in Canine Saliva: Is It Possible to Use a Commercial ELISA Kit?

**DOI:** 10.3390/ani9070418

**Published:** 2019-07-04

**Authors:** Jara Gutiérrez, Angelo Gazzano, Beatrice Torracca, Valentina Meucci, Chiara Mariti

**Affiliations:** Dipartimento di Scienze Veterinarie, Università di Pisa, 56124 Pisa, Italy

**Keywords:** blood, dogs, prolactin, saliva, stress

## Abstract

**Simple Summary:**

Prolactin is considered a remarkable index of stress response, both acute and chronic, in several species. Some studies have investigated the possibility of measuring prolactin in saliva in human beings and Rhesus macaque. The possibility of measuring it in dog saliva would provide a non-invasive, helpful tool for the assessment of dog welfare. The aims of this research article are to study (1) the possibility of quantifying canine prolactin in saliva using a prolactin canine ELISA (enzyme-linked immunosorbent assay) kit validated for measuring prolactin in canine blood and (2) the potential presence of a correlation between prolactin levels in saliva and plasma.

**Abstract:**

Prolactin has been reported to be a remarkable index of stress response, both acute and chronic, in several species. The use of biological matrixes other than blood is receiving increasing interest in the study of hormones, due to the lower invasiveness in collection. This research aimed to investigate the possibility of using a commercial ELISA (enzyme-linked immunosorbent assay) kit for measuring canine prolactin in blood for the quantification of canine prolactin in saliva. Study 1 consisted of a validation protocol, using saliva samples collected from lactating and non-lactating dogs. Study 2 was conducted to investigate a possible correlation between prolactin concentration in saliva and plasma in sheltered dogs by using the same kit. Prolactin values were reliably read only when they came from blood samples, not from saliva, but tended to be low in most of the cases. Study 1 showed that saliva had a matrix effect. In study 2, saliva prolactin levels were low and in 42.9% of cases, not readable. No correlation between prolactin values in plasma and saliva was found (ρ = 0.482; *p* = 0.274). These findings suggested that the determination of prolactin in dog saliva through an ELISA kit created for measuring prolactin in dog blood was unreliable.

## 1. Introduction

The best-known role of prolactin in the dog is the stimulation of the growth of the mammary gland and the lactation processes. Nevertheless, prolactin has over 300 separate biological activities and it plays multiple homeostatic roles and physiological functions in the organism, such as the electrolyte balance, luteal function, regulation of the immune system, osmoregulation, angiogenesis, maintenance of the inter-oestrous interval, etc. [1].

In addition, prolactin is considered an index of acute stress in some species. For instance, prolactin concentrations increased after various stressful stimuli in humans [2] and rats [3,4,5,6], including male rats [7]. In lactating rats, hyperprolactinemia seemed to be a significant factor for the decrease of plasma oxytocin response to acute stress [8]. Furthermore, prolactin seems to reduce anxiety-related behavior in both female and male rats, maybe because prolactin acts as an endogenous anxiolytic, both in males and non-pregnant female rats [9]. The decrease in stress-induced secretion of ACTH from the adenohypophysis and reduced corticosterone secretion from the adrenal gland in lactating rats is also manifested in mid-gestation, from day 15 until day 21 of pregnancy [10].

In human beings, prolactin-releasing stimuli include suckling, perception of visual, acoustic, and olfactory stimuli and stress [11]. For instance, it has been suggested that human serum prolactin concentrations may be elevated by psychological stressors, as well as by psychosocial stress (Trier Social Stress Test) [12]. In addition, in human beings, it is known that prolactin is in response to severe experimental stress induced by hypoglycaemia [2], surgery [2], parachute jumping in military recruits [13], and compulsory swimming in non-swimmers [14]. A significant correlation was found between day-to-day changes in anxiety measured by questionnaires as well as by stress hormones, cortisol, and prolactin [15]. 

Prolactin also increases in response to psychological stressors like restraint and transport in rats [16], heat stressin both rats and domestic ruminants [16,17], and stressful situations in donkeys [18], dromedaries [19], cattle [20], and sheep [21].

In dogs, both cortisol and prolactin decrease immediately after parturition [22]. Moreover, prolactin increases just after delivery due to the pups’ suckling stimulation [22]. In lactating bitches, high levels of prolactin and increased expression of prolactin receptors in the paraventricular nucleus may produce a decrease in the stress response during lactation [23,24]. In addition, anxious dogs displaying signs such as stereotypes, displacement activities, various autonomic disorders, and fear aggression, have an increase in prolactin blood levels [25]. Assistance dogs seem to have higher mean prolactin blood levels than pet dogs, suggesting a possible role of canine blood prolactin as an index of stress-related responses in dogs [26].

When quantifying certain physiological parameters, blood has often been used as the best body fluid to evaluate different biomarkers. However, in recent years, the replacement of blood by saliva samples has achieved growing interest due to its reduced invasiveness, cost, and risk of infection compared to blood collection [27]. Nevertheless, the concentration of these specific biomarkers may often differ between blood and saliva [28]. When multiple human biomarkers were compared in plasma and saliva samples, consistent correlations were found between both types of saliva sampling (passive drool and filter paper), but little correlation was found between plasma and saliva [28]. Korot’ko and Gotovtseva [29] found that human prolactin levels were lower in saliva than serum. In other cases, some methods were reported to be unable to quantify a certain biomarker in the saliva matrix, such as it being reported for salivary oxytocin when measured by immunoassay [30].

As prolactin is usually measured in blood to reduce the stress of blood sampling, it would be useful to find different biological matrixes in which prolactin could be reliably measured. The possibility of quantifying prolactin in human saliva has been evaluated using four commercially available methods different from ELISA (enzyme-linked immunosorbent assay), but none of which could detect it [31]. Salivary prolactin has instead been successfully measured in Rhesus macaques (*Macaca mulatta*) by radioimmunoassay, but a positive correlation between serum and salivary prolactin was not found, perhaps because the pulsatile variation of prolactin in the blood may be an impediment to detecting such a correlation [32]. This pulsatile secretion for prolactin in blood has also been found for dogs [33].

The aim of this study was to evaluate whether a commercially available ELISA kit for measuring canine prolactin in blood is also suitable for measuring canine prolactin in saliva. To do that, the research was divided into two parts: Study 1) consisted of a validation protocol using saliva samples collected from lactating and non-lactating dogs, and study 2 investigated the possible correlation between prolactin concentration in saliva and plasma in sheltered dogs.

## 2. Materials and Methods 

The procedure was communicated to the Ethics Committee of the University of Pisa, Italy (OPBA, Organismo Preposto per il Benessere Animale) and received a favorable opinion with Decision N.09/2018.

### 2.1. ELISA Kit

The prolactin ELISA kit used in this study is an enzyme immunoassay for the detection of canine prolactin in serum developed by Demeditec Diagnostic GmbH (Kiel, Germany). For this research, 2 kits were used and the assay was performed according to the manufacturer’s instructions. 

The microplate was coated with a monoclonal antibody specific for canine prolactin. Calibrators and samples were placed in front of the 96-well plate. A total of 25 μl calibrators and samples were added in triplicate in successive wells and incubated for 2 hours at room temperature. Endogenous canine prolactin in the sample binds to the antibodies fixed on the inner surface of the wells. Non-reactive sample components were removed by a washing step. 

Afterwards, a second polyclonal horseradish peroxidase-labeled antibody, directed against another epitope of the prolactin molecule, was added. During an hour of incubation, a sandwich complex consisting of the 2 antibodies and the canine prolactin was formed. After incubation, the plate was washed with the provided wash buffer and a substrate solution (3, 3′, 5, 5′-tetramethylbenzidine) was added, followed by another 30 min of incubation time. Finally, stop solution (hydrochloric acid) was added and the absorption was measured at 450 nm within 30 min with a Multiskan™ FC Microplate Photometer (ThermoFisher Scientific, Waltham, MA, USA). Concentrations of prolactin were estimated from a calibration curve obtained by plotting the optical density versus the concentration for each one of the calibrators (80.0, 40.0, 20.0, 10.0, 5.0, and 2.5 ng/ml).

### 2.2. Saliva and Plasma Samples Collection

For study 1, saliva samples from 21 healthy adult dogs (7 males and 14 females in anoestrus, 1–11 years old) were collected and pooled to establish a sample with regular values (non-lactating saliva, NLS). Saliva samples from 5 bitches in a lactation period (1–7 years old) were collected and pooled to establish a sample with assumed high levels of prolactin (lactating saliva, LS). Saliva was always collected in the morning by the same person, except for some lactating females, whose samples were collected by the person in charge. Saliva was collected using flat cottons (Salivette® Cotton swab, neutral 51.1534, Sarstedt) and gloves, in order to avoid variability in the results and contamination. Samples remained in a cold chain (0 to +4 °C) until they reached the laboratory, where they were centrifuged two consecutive times (7000 rpm, 10–15 min). Saliva was obtained after centrifugation and stored at −20°C until analysis.

For study 2, saliva and plasma samples were collected from 10 healthy adult mixed breed dogs (1 female and 9 males, 1–11 years old) single-housed in a municipal shelter, between 11:00 and 12:00 a.m. and always followed the same order: First blood, and then saliva, with an interval no longer than 2 minutes between them. With the exception of 3 dogs, from which we did not get enough saliva, both saliva and blood were analyzed for each dog. The extraction of saliva was carried out following the same protocol described for study 1. 

### 2.3. Validation Parameters

The possible application of an ELISA kit for canine blood prolactin to canine saliva samples (study 1) was determined by evaluating the linearity, limit of quantification, matrix effect, and spiking recovery. 

The lyophilized master calibrator (80 ng of lyophilized in serum/buffer matrix containing highly purified canine prolactin) was reconstituted with 1 ml of distilled water. To evaluate linearity, this prolactin standard solution was diluted with the provided sample diluent to obtain the solutions for the calibration curves (40.0, 20.0, 10.0, 5.0, and 2.5 ng/ml).

Previous literature shows that in dogs, blood prolactin levels tend to be low. In addition, unpublished data obtained by the authors of the current study showed that salivary prolactin levels are commonly low. Consequently, for this validation method, linearity could not be reliably assessed with serial dilutions of normal samples. In order to reach higher values that could improve the reading of the kit, it was decided to dilute standard solutions to obtain different final-added prolactin concentrations in artificial saliva (AS_20_, AS_10_, AS_5_, AS_2.5_; Pickering Laboratories^©^, Space Park Way, Mountain View, CA, USA),and in non-lactating saliva (NLS_20_, NLS_10_, NLS_5_). Parallelism between the curves obtained with standard solutions and the ones obtained with the spiked saliva pool was also assessed. 

Lactating saliva pool (LS), with assumed high prolactin concentration, was diluted (2:3 and 1:2 for kit 1; 2:3 for kit 2) using artificial saliva as a diluent to evaluate linearity. The goal of analyzing lactating dog saliva as such (LS) and in two different dilutions (LS_d1_, LS_d2_) was to find out the minimum concentration of salivary prolactin that could be read by the kit.

To assess the possible matrix effect and recovery for each kit, 3 repetitions of a lyophilized control from the same batch (Demeditec Diagnostics GmbH^®^, Kiel, Germany) were reconstituted (1 ml) with water (control reconstituted in water = CW; as specified by the manufacturer instructions), AS (control reconstituted in artificial saliva = CAS),and NLS (control reconstituted in saliva of non-lactating dogs = CNLS). For the first kit, 3 repetitions for each reconstitution were made using a control batch corresponding to 15.14 ng/ml, and for the second kit, a control batch of 8.16 ng/ml was used (Appendix A).

The lower limit of sensitivity was determined as the mean concentration obtained interpolating the optical density plus 2 SDs for all replicates of the 0 standard.

### 2.4. Statistical Analysis

The possible presence of correlation between the prolactin values in blood and in saliva for study 2 has been analyzed through the Spearman’s Rho test (*p* < 0.05).

## 3. Results

Linearity was acceptable for all the regression curves for prolactin concentrations in sample diluent, in AS and in NLS, for both kits (R^2^ values: Sample diluents kit 1 = 0.995, AS kit 1 = 0.99, NLS kit 1= 0.994; sample diluent kit 2= 0.996, AS kit 2 = 0.992, NLS kit 2 = 0.993). Slopes between these regression curves were parallel for each kit, respectively (sample diluent kit 1 = 0.033, AS kit 1 = 0.034, NLS kit 1 = 0.037; sample diluent kit 2 = 0.032, AS kit 2 = 0.035, NLS kit 2 = 0.037) and R^2^ coefficients for the same curves were acceptable for both kits, meaning that kits ran similarly.

Levels of prolactin measured in non-lactating (NLS) and lactating (LS) saliva pools were similar and, in both cases, low (mean ± standard deviation: NLS = 0.34 ± 0.30 ng/ml; LS = 1.03 ± 1.22 ng/ml for kit 1; NLS = 1.07 ± 0.20 ng/ml; LS = 1.17 ± 0.07 ng/ml for kit 2). Due to these low levels, it was not possible to conduct a linearity analysis based on physiological sample dilutions. 

Values for the non-lactating saliva pool with the addition of the standards to obtain different final added concentrations (kit 1: NLS_20_ = 23.4 ng/ml, NLS_10_ = 11.4 ng/ml, NLS_5_ = 6.4 ng/ml; kit 2: NLS_20_ = 24.1 ng/ml, NLS_10_ = 12.3 ng/ml, NLS_5_ = 6.4 ng/ml) were higher than the normal added prolactin concentrations (20, 10, and 5 ng/ml).

Prolactin concentration values obtained in CW, CAS, and CNLS (Table 1) were within the target ranges, except for CNLS in kit 2 which was higher (12.1 ng/ml). CAS obtained virtually the same value than CW in both kits. Prolactin concentration values in CNLS were slightly higher than CW, in both kits (kit 1: CNLS–CW = 18.7–16.5 = 2.2 ng/ml; kit 2: CNLS – CW = 12.1–9.7 = 2.4 ng/ml). Values of CNLS were slightly higher than CAS (Table 1). 

The lower limit of sensitivity was calculated as the mean concentration obtained interpolating the optical density plus 2 SDs for all replicates of the 0 standard was 1.10 ng/ml.

A reliable inter-assay repeatability was recorded for values whose concentrations were higher than 5 ng/ml (e.g., AS_20_, AS_10_, AS_5_, F_10_, F_5_), with coefficients of variation lower than 12% (mean %CV ± standard deviation = 8.13% ± 0.03), whereas for lower concentrations (e.g., AS_2.5_, LC, LC_Dil1_, F_20_, F), high coefficients of variation were recorded (mean %CV ± standard deviation = 71.54% ± 0.32).

Prolactin concentration values in saliva and plasma samples obtained in study 2 are shown in Table 2. Prolactin values from plasma samples were below the limit of the kit’s detection (0.4 ng/ml) in 20.0% of cases (mean ± standard deviation = 4.69 ± 5.37 ng/ml), and were especially high for dog 1 (17.8 ng/ml). Saliva prolactin levels were low too (mean ± standard deviation = 1.94 ± 1.96 ng/ml) and, in 42.9%of cases, not detectable. In fact, prolactin concentration in saliva samples without any additions resulted in very low values in both study 1 and 2.

Values from Table 2 clearly show that there is no correspondence between prolactin concentrations in plasma and in saliva: On one hand, dogs 1, 3, and 7 had the highest values of plasma prolactin, but low saliva concentrations, and on the other hand, dog 4 had a relatively high value of saliva prolactin, but prolactin in plasma was not even detectable. This was confirmed by a lack of correlation between plasma and saliva values (ρ = 0.482; *p* = 0.274).

## 4. Discussion

Both kit 1 and 2 were able to read the control within the optimal target ranges. Artificial saliva (AS) did not seem to interfere with the reading of the kit, as the reading of control joined to artificial saliva (CAS) was almost the same for the reading for control added to water (CW). In other terms, artificial saliva did not seem to have a matrix effect. However, saliva is a complex matrix and for this reason further measurements were done using real saliva, which will be discussed later. 

In both studies, prolactin concentration in saliva samples without any additions resulted in very low values, often below the limit of detection of the kit, meaning that the kit cannot feasibly read prolactin concentrations in natural saliva samples.

A difference emerged between the values of CW and the values of control joined to non-lactating saliva pool (CNLS). It could be hypothesized that this difference was due to the fact that the kit was reading the prolactin present in the non-lactating saliva pool. However, this does not seem to be a justifiable explanation, since the concentration of prolactin in the non-lactating saliva pool alone (NLS) was lower than the difference between CNLS and CW. Since NLS was used as an example of natural saliva, it can be deduced that such a difference was due to a matrix effect of the natural canine saliva itself, and consequently the kit could not read saliva samples properly. Sometimes, a matrix effect can disappear by diluting the sample serially until a linearity of the results is obtained. However, serial dilutions of the lactating saliva pool (LS) did not exhibit a linear dilution, indicating that a matrix component was interfering with an accurate detection, causing a loss in reading sensitivity.

The variation between the values for non-lactating saliva pool with addition of the standards to obtain different final added concentrations (NLS_20_, NLS_10_, NLS_5_) and those of the corresponding standards (20, 10, and 5 ng/ml) are not due to the presence of natural prolactin, since calculated NLS values were different when using the different additions. For this reason, natural prolactin could not be reliably determined with the addition of the standards and the observed difference was likely not reflecting the real value of prolactin, due to the presence of a matrix effect of the saliva. In other terms, although the addition of the prolactin standard allowed higher values to be obtained and, therefore, a reading of prolactin results feasible, a large matrix effect was observed, preventing the assertion that the value obtained was reliable. 

Furthermore, we expected prolactin concentrations in non-lactating saliva to be lower than prolactin concentration of saliva samples in lactating dogs, since prolactin levels in blood have been reported to have an increase in lactating dogs [33,34,35]. Indeed, saliva from lactating dogs had values that were only slightly higher than those of non-lactating dogs and with a high variation. In addition, the reliability of NLS and LS readings was not adequate because their coefficients of variation were quite high. 

In most of the cases, the kit was not able to detect prolactin in saliva samples as obtained values were very low and some of them did not reach the limit of the kit’s detection. In study 2, when prolactin in saliva was detected by the kit, most of these values were higher than the ones from the lactating saliva pool, as mirrored by mean values. A possible explanation might be that shelter dogs are often exposed to stressful conditions, leading to higher prolactin circulating levels [2,14,15]. However, the lack of correlation with plasma concentrations did not allow us to draw reliable conclusions. 

In study 2, plasma prolactin concentrations, when readable, agreed with values reported by other authors for canine prolactin in serum, ranging 1–6.3 ng/ml [36,37,38,39]. One exception in the current study is represented by dog 1, with high plasmatic prolactin values, probably due to its behavioral problems since it showed a phobia of numerous stimuli, and possibly a state of anxiety (not assessed as it was out if the scope of this study). The relationship between phobia, anxiety, and prolactin has been investigated by Pageat, 2007 [25], however further research is needed for a better understanding of their possible links. 

A correlation between plasmatic and salivary prolactin levels from paired single samples was not found. This fits with the results of Lindell et al. [32], who did not find a positive correlation between values of prolactin in the serum and saliva of Rhesus macaques, discussing the possibility that the pulsatile variation of prolactin in serum impedes the detection of a significant relationship with the pooled saliva source, since single blood samples were used, eventually suggesting that a repeated blood sampling might lead to a significant correlation [32].

In this study, blood and saliva were only collected from non-lactating stressed dogs at the shelter, while for the two pools used for validation (lactating dogs and non-lactating dogs), individual samples were not collected. Although having the results of blood prolactin levels from the same samples of saliva used for the validation assay would have been optimal, blood collection was avoided in lactating bitches for ethical reasons. We also avoided collecting multiple saliva samples from lactating bitches, in order to minimize interference with their nursing and lactation. Due to the low values obtained in study 1 for both non-lactating and lactating dogs, for study 2, it was decided to use individual samples of blood and saliva from sheltered dogs, which possibly had higher prolactin values due to stress.

Moreover, the use of Salivette for collecting saliva samples may have caused a bias in the results. The different available saliva collection methods were reported to possibly interfere in the biomarker of interest present in the saliva. For this reason, in certain cases, a correspondence between methods of collection was found. For example, Salivette was reported to be a reliable and predictable method of total and quantified free serum cortisol levels [40]. However, in other cases this correspondence was not found. For instance, nephelometrically determined IgA concentrations were significantly lower in saliva when collected by the Salivette than by a suction or spitting method [41]. When quantifying cortisol and dehydroepiandrosterone (DHEA), results using two different saliva collection methods (‘passive drool’ method and a citric acid-treated salivette) correlated highly with plasmatic levels and with each other, whereas results using Salivette did not correlate significantly with plasma values [42]. Future research using other methods of saliva collection may be useful for knowing if the method of collection interferes with the measurement of prolactin in saliva.

Taken together, all these results suggest that prolactin cannot be reliably measured in saliva by the ELISA kit used in the study, which is validated for measuring prolactin in dog blood, meaning that canine saliva was not a suitable matrix for the kit. The quantification of prolactin though ELISA kits out of the intended use needs caution. In fact, a previous study found that prolactin in horses cannot be reliably measured using a canine prolactin ELISA kit, due to a matrix effect [43].

## 5. Conclusions

In summary, for saliva the study found: A matrix effect, very low prolactin concentrations (often under the limit of detection), and a lack of correlation with prolactin plasmatic levels. Validation of the kit showed that prolactin in saliva could be read under certain conditions (standard addition) but without reliability (matrix effect). In the absence of standard addition, prolactin values were too low to be read.

These results suggest that saliva was not a suitable matrix to measure prolactin levels using an ELISA kit created for measuring canine blood prolactin concentrations. 

## Figures and Tables

**Table 1:** Expected and observed prolactin values when control was dissolved in water (CW), artificial saliva (CAS), and non-lactating saliva (CNLS). Mean and standard deviation for the observed values and recovery percentage.

Sample		Expected: Mean (ng/ml)	Expected: Target Range (ng/ml)	Observed (ng/ml)	CV	Recovery
Control dissolved in water (CW)	Kit 1:	15.14	9.1–21.2	16.5 (s.d. 0.45)	2.8%	109% (s.d. 3)
	Kit 2:	8.16	4.9–11.4	9.7 (s.d. 0.21)	2.2%	119% (s.d. 3)
Control dissolved in artificial saliva (CAS)	Kit 1:	15.14	9.1–21.2	16.3 (s.d. 0.84)	5.2%	107% (s.d. 6)
	Kit 2:	8.16	4.9–11.4	9.9 (s.d. 0.59)	5.9%	122% (s.d. 7)
Control dissolved in non-lactating saliva (CNLS)	Kit 1:	15.14	9.1–21.2	18.7 (s.d. 1.09)	5.8%	124% (s.d. 7)
	Kit 2:	8.16	4.9–11.4	12.1 (s.d. 0.61)	5.0%	148% (s.d. 7)

**Table 2:** Prolactin concentrations in saliva and plasma samples (ng/ml) using a canine prolactin ELISA (enzyme-linked immunosorbent assay) kit. (BLD = below the limit of detection).

	Plasma		Saliva	
	Concentration (ng/ml)	Coefficient of Variation	Concentration (ng/ml)	Coefficient of Variation
Dog 1	17.8	1.3%	BLD	-
Dog 2	1.5	1.1%	BLD	-
Dog 3	3.6	1.9%	BLD	-
Dog 4	BLD	-	4.6	8.1%
Dog 5	2.6	2.6%	0.0	3.0%
Dog 6	2.1	1.6%	2.0	1.6%
Dog 7	4.5	1.8%	1.1	2.2%
Dog 8	BLD	-	-	-
Dog 9	2.8	1.5%	-	-
Dog 10	2.5	9.3%	-	-

## Appendix A

**Table A1:** Outline distribution adopted for the validation procedure. Details of the wells for kit 1 and kit 2. The only difference is that the second dilution for lactating female saliva (LS_dil2_) was replaced in kit 2 by saliva from a different pool of domestic dogs (saliva as such -P-, and two dilutions of it -P_20_ and P_10_-). Caption: A-G (reconstitute lyophilized canine prolactin master calibrators), CW (control dissolved in water), CAS (control dissolved in artificial saliva), CNLS (control dissolved in non-lactating dogs saliva), AS_20_-AS_2.5_ (artificial saliva with addition of the standards), LS (lactating dogs saliva pool), LS_d1_-LS_d2_ (dilutions of lactating dogs saliva pool), NLS (non-lactating dogs saliva pool), N_20_-N_10_-N_5_ (non-lactating dogs saliva pool with addition of the standards, respectively 20, 10 and 5 ng/ml), NLS+CNLS (non-lactating dogs saliva pool + control dissolved in saliva, 1:1).

**Kit 1**	**1**	**2**	**3**	**4**	**5**	**6**	**7**	**8**	**9**	**10**	**11**	**12**
A	A _0_	E _20_	CW	CNLS	AS_20_	AS_10_	AS_2.5_	LS	LS_d2_	N	NLS_20_	NLS_5_
B	A _0_	E _20_	CW	CNLS	AS_20_	AS_10_	AS_2.5_	LS	LS_d2_	NLS	NLS_20_	NLS_5_
C	B _2.5_	F _40_	CAS	AS	AS_20_	AS_5_	AS_2.5_	LS_d1_	LS_d2_	NLS	NLS_10_	NLS_5_
D	B _2.5_	F _40_	CAS	AS	AS_20_	AS_2.5_	AS_2.5_	LS_d1_	LS_d2_	NLS	NLS_10_	NLS_5_
E	C _5_	G _80_	CAS	AS	AS_10_	LS	LS	LS_d1_	LS_d2_	NLS_20_	NLS_10_	NLS_5_
F	C _5_	G _80_	CAS	AS	AS_10_	LS	LS	LS_d1_	LS_d2_	NLS_20_	NLS_10_	NLS_5_
G	D _10_	CW	CNLS	AS_20_	AS_10_	LS	LS	LS_d1_	NLS	NLS_20_	NLS_10_	NLS + CNLS
H	D _10_	CW	CNLS	AS_20_	AS_10_	LS	LS	LS_d1_	NLS	NLS_20_	NLS_10_	NLS + CNLS
**Kit 2**	**1**	**2**	**3**	**4**	**5**	**6**	**7**	**8**	**9**	**10**	**11**	**12**
A	A _0_	E _20_	CW	CNLS	AS_20_	AS_10_	AS_2.5_	LC	P	NLS	NLS_20_	NLS_5_
B	A _0_	E _20_	CW	CNLS	AS_20_	AS_10_	AS_2.5_	LC	P	NLS	NLS_20_	NLS_5_
C	B _2.5_	F _40_	CAS	AS	AS_20_	AS_5_	AS_2.5_	LS_d1_	P_20_	NLS	NLS_10_	NLS_5_
D	B _2.5_	F _40_	CAS	AS	AS_20_	AS_2.5_	AS_2.5_	LS_d1_	P_20_	NLS	NLS_10_	NLS_5_
E	C _5_	G _80_	CAS	AS	AS_10_	LS	LS	LS_d1_	P_20_	NLS_10_	NLS_10_	NLS_5_
F	C _5_	G _80_	CAS	AS	AS_10_	LS	LS	LS_d1_	P_10_	NLS_20_	NLS_10_	NLS_5_
G	D _10_	CW	CNLS	AS_20_	AS_10_	LS	LS	LS_d1_	NLS	NLS_20_	NLS_10_	NLS + CNLS
H	D _10_	CW	CNLS	AS_20_	AS_10_	LS	LS	LS_d1_	NLS	NLS_20_	NLS_10_	NLS + CNLS
